# King Edward's Hospital Fund for London Statistics
*The first notice appeared in The Hospital of October 10, p. 45.


**Published:** 1914-12-12

**Authors:** 


					HOSPITAL ECONOMICS.
King Edward's Hospital Fund for London Statistics.
rp. (Second Notice)
Ittt? *i  ^r il tt' ?
The compilers of the King Edward's Hospital
una '' Blue-book '' in marshalling their facts for
purpose of comparing the expenditure of hos-
j^al with hospital have arranged the institutions
^ groups. We are, therefore, able to examine side
^ side the statistics of the larger general, the
'jailer general, cottage and lying-in hospitals, and
le hospitals for consumptives, women, children,
u sufferers from ophthalmic and nervous dis-
f^ses. Tlie arrangement has something to be said
* J?r> and something against it. To have the figures
the great general hospitals separated from
r. aller and miscellaneous institutions, and scienti-
? Ca% analysed and compared, is instructive in
.Sejf and helpful in comparing the results with
^llar information respecting other hospitals.
Right and Wrong Ways of Grouping.
Ohi^Uk ^le h?sP^a^s in the groups of consumption,
t. ^nalmic, and nervous disease hospitals are as
be comParison within the group as would
dp f3, ?Dreadnought in company with one or two
s hyers and smaller craft. The inherent diffi-
y of comparing a general with a special hospital
fiot ^ean*n& lessons from the result is really
th -?r?a^er than the same process applied to, say,
Pit ] .nce "Wales's and the Metropolitan Hos-
ag , s> institutions of equal size, but widely differing
side? ^.rac^ion, locality, and other influential con-
Iftions. In any case it would be instructive to
5arri le three special groups mentioned above in the
be 6 table, and in support of this suggestion it may
^^sntioned that the three chief hospitals con-
cerned ai^e no further apart from the financial point
of view, as illustrated by the cost per occupied bed,
than any three general hospitals selected at random
from the other fables.
COMPABISON AND 'ECONOMY.
It will be remembered that the comparisons are
limited to items of expenditure which are, within
limits, easily capable of control. Outlay on repairs
to and upkeep of buildings, official salaries and pen-
sions, bank charges, appeals, rent, rates and taxes,
while shown in certain tables, are excluded from
those forming a basis for the calculation of com-
parable figures as to the cost per occupied bed and
per out-patient attendance. This is, of course, not
to say that economy is less essential under the
excluded headings than under the others, the
arrangement simply makes a gener-al allowance for
the fact that the management of a hospital cannot
help, or easily rectify, disadvantages caused by the
age of the building, the pensionable claims of its
staff, and other matters widely differing between
institutions roughly comparable in other respects.
We can, therefore, regard our statistics unembar-
rassed by larger variable factors, although we have
to bear in mind at all times, however ingeniously
hospital statistics are arranged, that there are
always considerations that, as we hope to show,
have a constant tendency to control the ebb and
flow of expenditure.
Since the advent of the new forms of accounts, as
adopted at the instance of the three Central Funds
in 1908, six sets of comparative tables have been
The first notice appeared in The Hospital of Octo ber 10, p. 45.
246 THE HOSPITAL December 12, 1914-
issued by King Edward's Hospital Fund. The
impetus to economy given by the interchange
of information, originated in 1903, was in-
creased by the more satisfactory method of
keeping accounts advised by Mr. J. G. Griffiths,
and had very visible results up to 1911. That it is
still a potent instrument for good is not disproved
by the fact that the abnormally expensive years
1912-1913 (and probably, alas! 1914) have not
brought a further decrease in the cost per occupied
Table I.
Seventeen Larger General Hospitals Arranged to Show Relative Positions in Respect of Cost pef.
Occupied Bed and per Out-patient Attendance for Six Years.
(Xote : 2nd = second lowest, 3rd = third lowest, etc.)
Charing Cross
German
Great, Northern Central
Guy's
King's College
London
Metropolitan
Middlesex
Prince of Wales's ...
lloyal Free
St. George's
St. Mary's
St. Thomas's
Seamen's
University College
West London
Westminster
1908
In
Patient
16th
3rd
13th
5th
12th
14th
11th
9th
Lowest
10th
Highest
6th
15th
2nd
7th
4th
8th
Out
Patient
13th
5th
6th
Lowest
Highest
11th
10th
9th
4th
15th
8th
7th
14th
3rd
16th
2nd
12th
1909
Iri
Patient
15th .
4th
12th
7th
13th
14th
Closed
5th
Lowest
10th
Highest
8th
11th
2nd
6th
3rd
9th
Out
Patient
13th
5th
7th
Lowest
Highest
llth
Closed
6th
3rd (tie) I
15th
9 th
8th
14th.
3rd (tie)
12th
2nd
10th
1910
Ill
Patient
14th
5th
15th
7lh
13th
Highest
8th
9th
2nd
11th
No re
6th
12th
Lowest
4th
3rd |
10th
Out
Patient
12th
6th
fth
Lowest
Highest
14th
8th
5th
4th
15th
turn
7t,h
13th i
3rd
11th J
2nd
10th
1911
In
Patient
12th
Closed
7th
6th
13th
9th
8th
15th
2nd
11th
Highest
bth
10th
Lowest
4th
3rd
14th
Out
Patient
11th
Closed
7th
2nd
Highest
10th
6th
8th
3rd
15th
9th
5th
14th
4th
13th
Lowest
12th
1912
In
Patient
14th
Closed
6th
7th
If tli
11th
13th
5th
2nd
12th
Highest
8th
9th
Lowest
3rd
4th
10th
Out
Patient
14th
Closed
7th
2nd
Highest I
13th
4th
6th
3rd
15th
8th
10th
11th
5th
12th
Low est
9th
1913
In
Patient
11th
5th
7th I
10th
No re
lfth
9th !
6th
2nd
13th i
Highest 1
8ih
14th
Lowest
4th
3rd |
12th
Out
Patie?
9th
3rd
7th
2m1
turn
13th
6tb
:th
Lotff'
ICtb
lltb
]5tn
I4tii
8tb
High?5*
4th
12tb
M = Mcdical School.
bed and per out-patient attendance. The issue of
the statistics enables us to do two things: the first
is to study the figures of a single year; and the
second, which we venture to think is sometimes
overlooked, is to compare year with year. As an
instance of the possibilities of the latter facility
we have prepared the accompanying table, showing
which hospitals have held their own in the fight
for economy, and which have gained or lost ground.
We are able by means of the foregoing table to
rank the seventeen hospitals concerned in their
average position over the period covered, and, for
convenient reference, we have placed side by side
the position resulting from the calculations for 1913.
Table II.
Cost per Occupied Bed
Cost per Out-patient Attendance
Seamen's
Prince of Wales's
West London ...
German
(five years only)
University Coll. M
St. Mary's M
Guy's ... M
Middlesex M
Grett Northern
Central
Westminster M
Royal Free M
Metropolitan
(five years only)
St. Thomas's M
King's College M
London ... M
Charing Cross M
St. George's M
(five years only)
1908 to
1913
Lowest
2nd
3rd
4th
5th
6th
7th
8th
9th
10th
11th
12th
13th
\14th
/(tie) |
16th 1
Highest:
1913
Lowest
2nd
3rd
5th
4th
8th
10th
6th
7th
12th
13th
9th
14th
No
return
15th !
11th I
Highest:
Guy's ... M
West London
Prince of Wales's
Seamen's
German ...
Middlesex
Metropolitan
Great Northern
Cpntral
St. Mary's
St. George's
Westminster
Charing Cross ..
London ...
University Coll.
St. Thomas's
Royal Free
King's College...
1908 to
1913
Lowest
2nd
3rd
4th !
5th
6th
7th
8th
9th
10th
11th
12th
13th
14th
15th
16th |
Highestl
1913
2nd
4th
Lowest
8th
3rd
5th
6th
7th
15th
11th
12th
9th
13 th
First Survey of the Tables : A Few
Interesting Points.
A first survey of these tables reveals several ma111
facts. One is the close competition between three oat-
lying hospitals for pride of place in both in-patie1^
and out-patient expenditure; the consistent places o1
St. George's and King's College Hospitals as most
expensive respectively under the cost of the
and the attendance; the curious discrepancy in til?
position of University College in the in- and oat'
patient columns, and the extraordinary fluctuati?'j
of the Middlesex, the Great Northern Central, 311
some other hospitals from year to year.
We hope to examine these matters in gre ,e
detail in a future article, and, in considering \
advantages and disadvantages of a "flat rate ' 1
calculating out-patienfc expenditure, to again tab
late the greater hospitals, and possibly some otl^1 ^
after the statistics have been rearranged on sue'1
basis.

				

